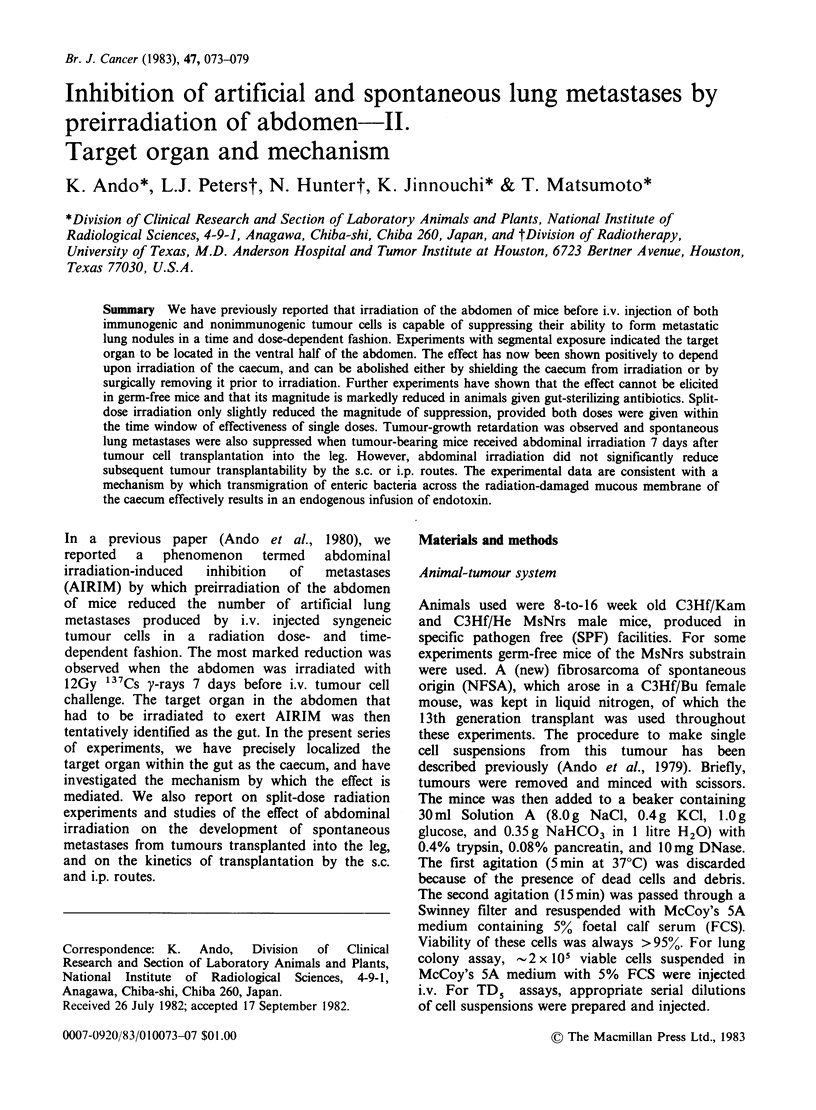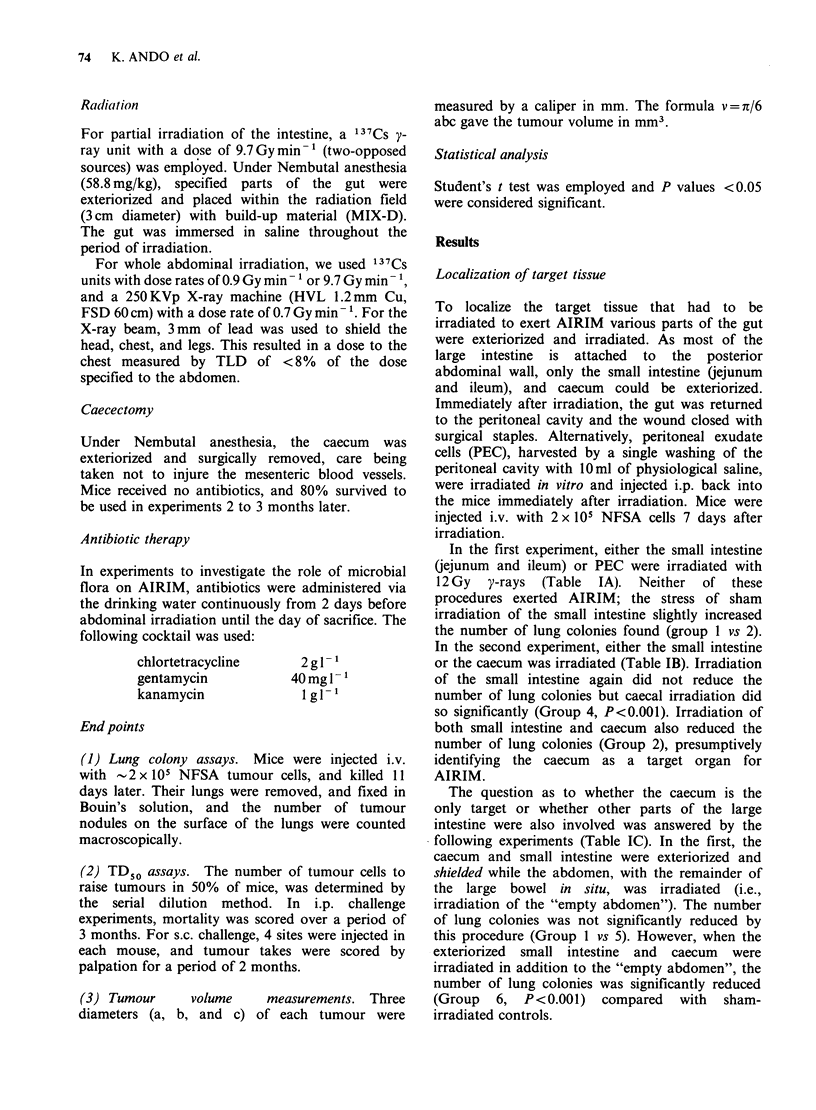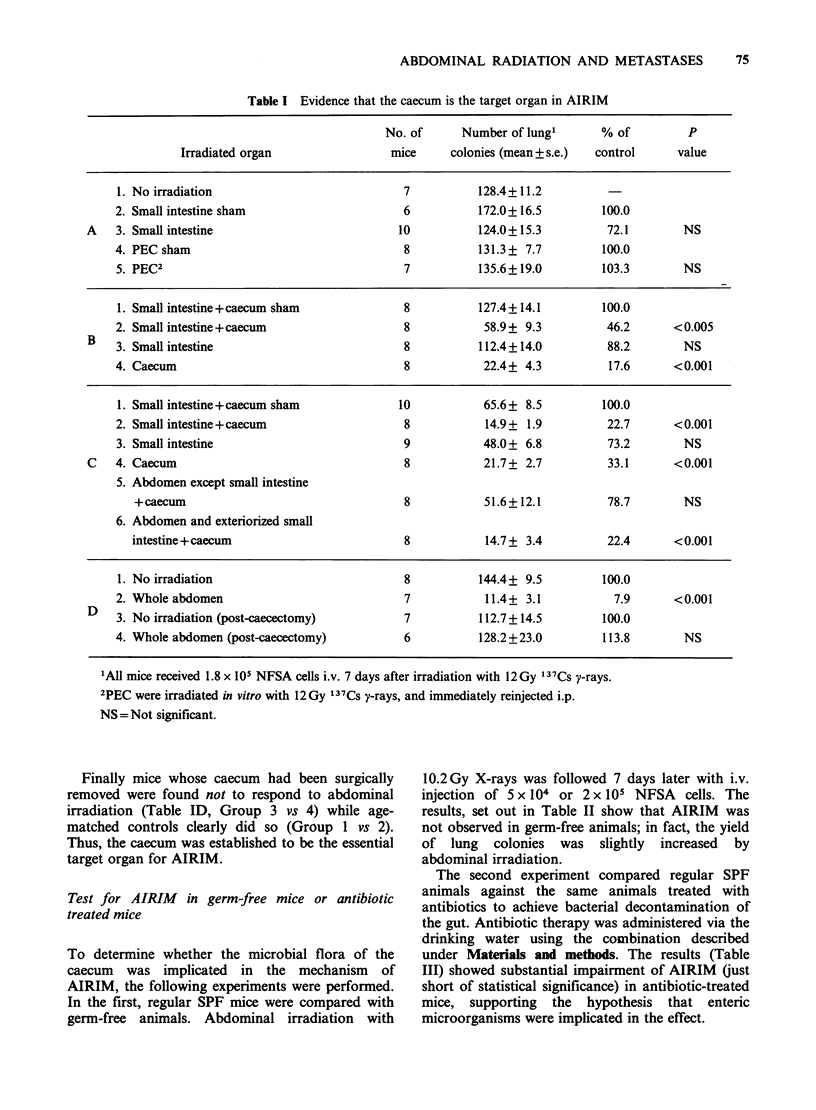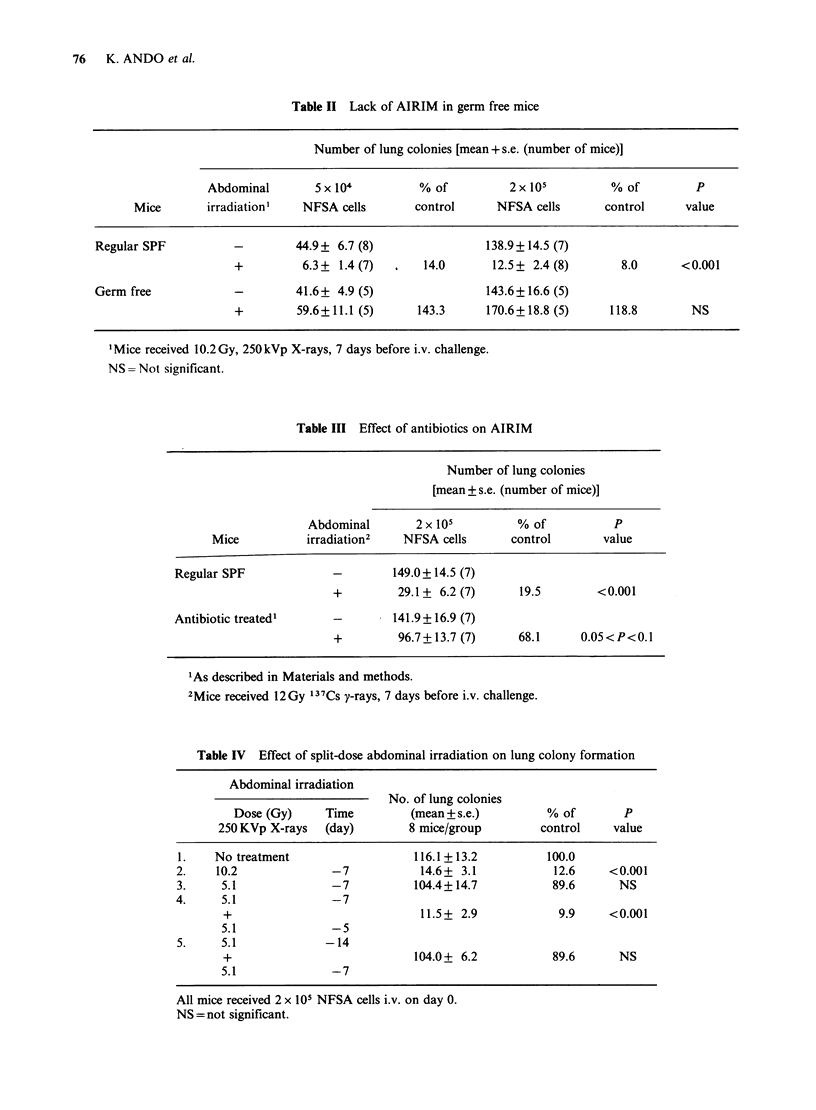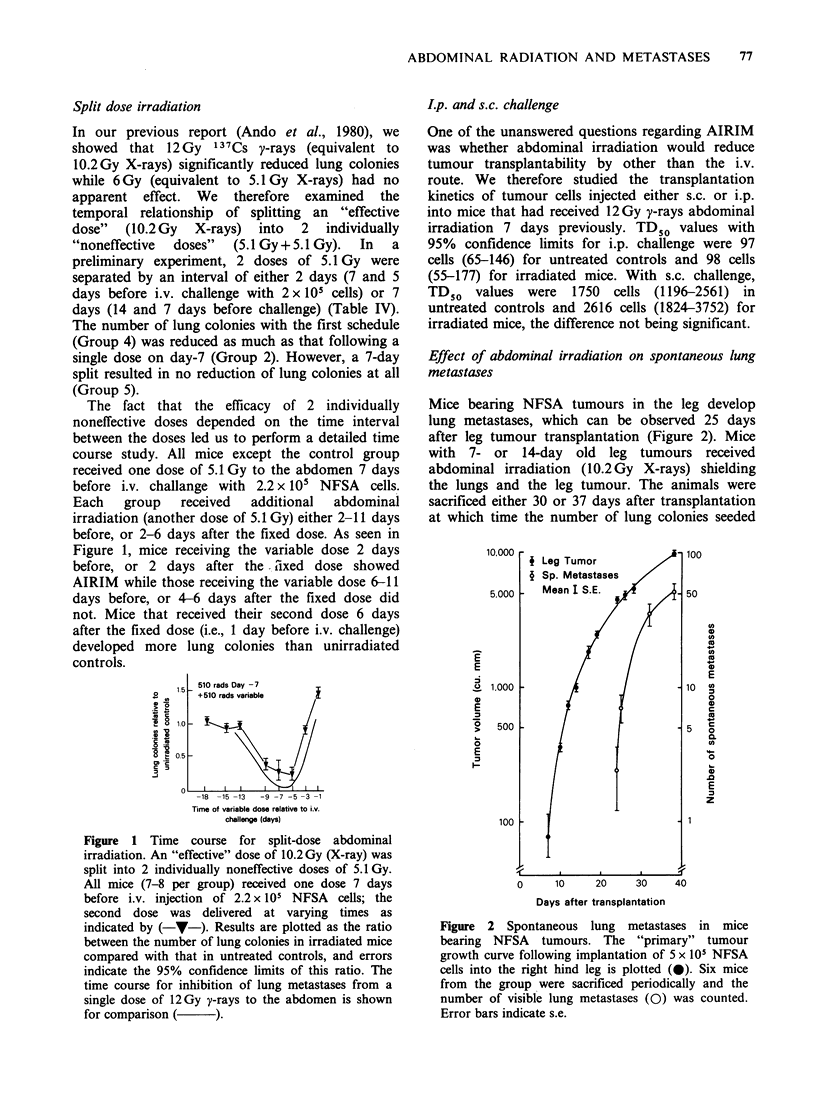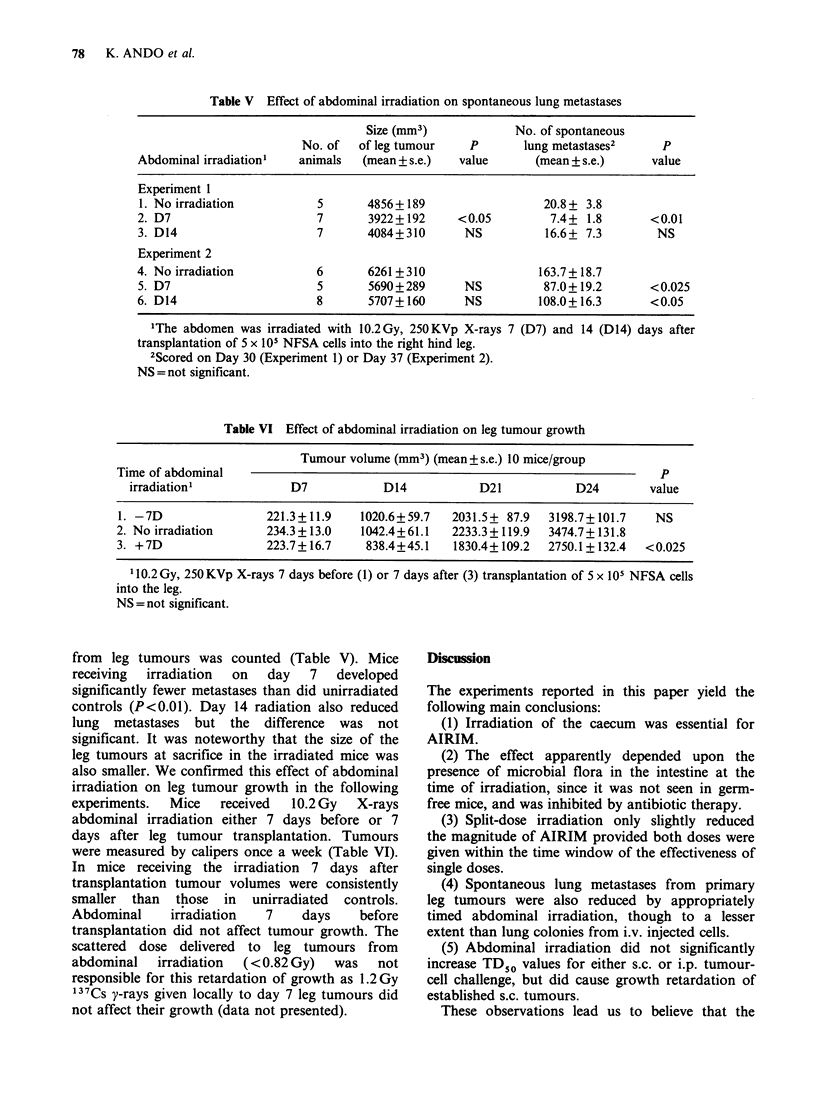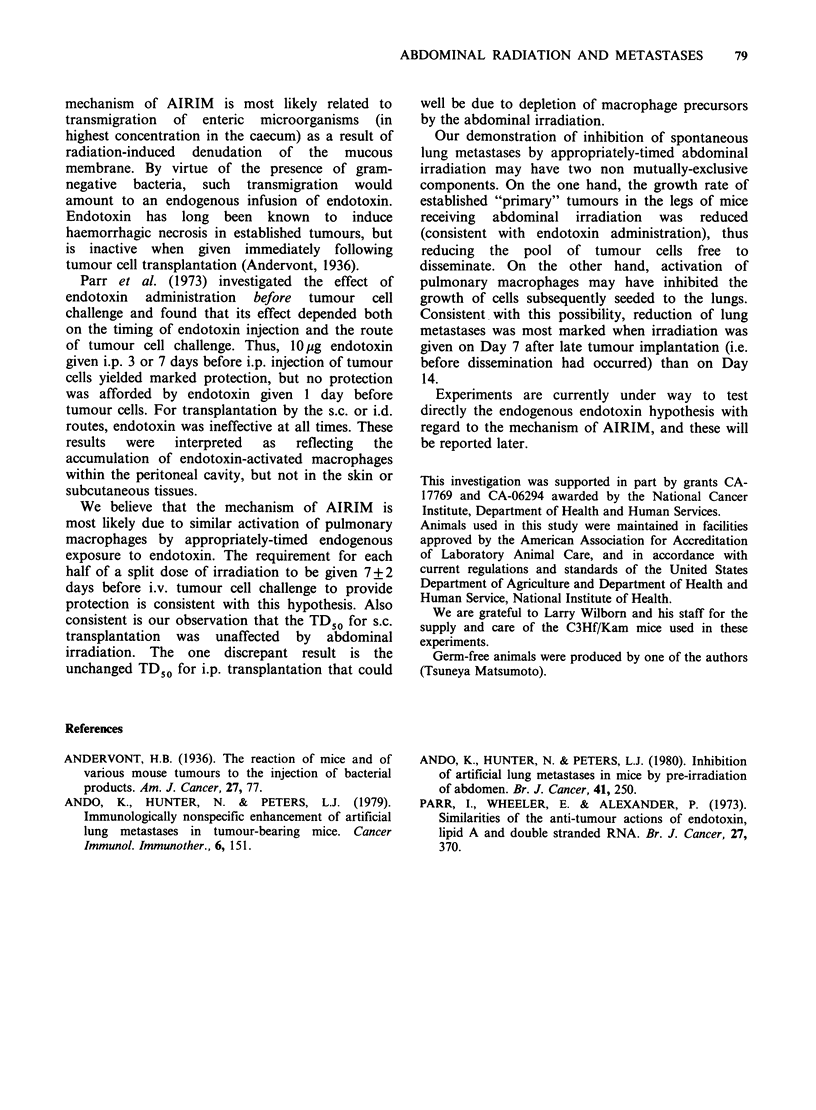# Inhibition of artificial and spontaneous lung metastases by preirradiation of abdomen--II. Target organ and mechanism.

**DOI:** 10.1038/bjc.1983.9

**Published:** 1983-01

**Authors:** K. Ando, L. J. Peters, N. Hunter, K. Jinnouchi, T. Matsumoto

## Abstract

We have previously reported that irradiation of the abdomen of mice before i.v. injection of both immunogenic and nonimmunogenic tumour cells is capable of suppressing their ability to form metastatic lung nodules in a time and dose-dependent fashion. Experiments with segmental exposure indicated the target organ to be located in the ventral half of the abdomen. The effect has now been shown positively to depend upon irradiation of the caecum, and can be abolished either by shielding the caecum from irradiation or by surgically removing it prior to irradiation. Further experiments have shown that the effect cannot be elicited in germ-free mice and that its magnitude is markedly reduced in animals given gut-sterilizing antibiotics. Split-dose irradiation only slightly reduced the magnitude of suppression, provided both doses were given within the time window of effectiveness of single doses. Tumour-growth retardation was observed and spontaneous lung metastases were also suppressed when tumour-bearing mice received abdominal irradiation 7 days after tumour cell transplantation into the leg. However, abdominal irradiation did not significantly reduce subsequent tumour transplantability by the s.c. or i.p. routes. The experimental data are consistent with a mechanism by which transmigration of enteric bacteria across the radiation-damaged mucous membrane of the caecum effectively results in an endogenous infusion of endotoxin.


*Br. J. Cancer* (1983), **47**, 073–079

### Inhibition of artificial and spontaneous lung metastases by preirradiation of abdomen—II. Target organ and mechanism

K. Ando*, L.J. Peters†, N. Hunter†, K. Jinnouchi* & T. Matsumoto*

**Division of Clinical Research and Section of Laboratory Animals and Plants, National Institute of Radiological Sciences, 4-9-1, Anagawa, Chiba-shi, Chiba 260, Japan, and †Division of Radiotherapy, University of Texas, M.D. Anderson Hospital and Tumor Institute at Houston, 6723 Bertner Avenue, Houston, Texas 77030, U.S.A.*

**Summary** We have previously reported that irradiation of the abdomen of mice before i.v. injection of both immunogenic and nonimmunogenic tumour cells is capable of suppressing their ability to form metastatic lung nodules in a time and dose-dependent fashion. Experiments with segmental exposure indicated the target organ to be located in the ventral half of the abdomen. The effect has now been shown positively to depend upon irradiation of the caecum, and can be abolished either by shielding the caecum from irradiation or by surgically removing it prior to irradiation. Further experiments have shown that the effect cannot be elicited in germ-free mice and that its magnitude is markedly reduced in animals given gut-sterilizing antibiotics. Split-dose irradiation only slightly reduced the magnitude of suppression, provided both doses were given within the time window of effectiveness of single doses. Tumour-growth retardation was observed and spontaneous lung metastases were also suppressed when tumour-bearing mice received abdominal irradiation 7 days after tumour cell transplantation into the leg. However, abdominal irradiation did not significantly reduce subsequent tumour transplantability by the s.c. or i.p. routes. The experimental data are consistent with a mechanism by which transmigration of enteric bacteria across the radiation-damaged mucous membrane of the caecum effectively results in an endogenous infusion of endotoxin.

In a previous paper (Ando *et al.*, 1980), we reported a phenomenon termed abdominal irradiation-induced inhibition of metastases (AIRIM) by which preirradiation of the abdomen of mice reduced the number of artificial lung metastases produced by i.v. injected syngeneic tumour cells in a radiation dose- and time-dependent fashion. The most marked reduction was observed when the abdomen was irradiated with 12Gy $^{137}Cs$ $\gamma$-rays 7 days before i.v. tumour cell challenge. The target organ in the abdomen that had to be irradiated to exert AIRIM was then tentatively identified as the gut. In the present series of experiments, we have precisely localized the target organ within the gut as the caecum, and have investigated the mechanism by which the effect is mediated. We also report on split-dose radiation experiments and studies of the effect of abdominal irradiation on the development of spontaneous metastases from tumours transplanted into the leg, and on the kinetics of transplantation by the s.c. and i.p. routes.

Correspondence: K. Ando, Division of Clinical Research and Section of Laboratory Animals and Plants, National Institute of Radiological Sciences, 4-9-1, Anagawa, Chiba-shi, Chiba 260, Japan.
Received 26 July 1982; accepted 17 September 1982.

### Materials and methods

### *Animal-tumour system*

Animals used were 8-to-16 week old C3Hf/Kam and C3Hf/He MsNrs male mice, produced in specific pathogen free (SPF) facilities. For some experiments germ-free mice of the MsNrs substrain were used. A (new) fibrosarcoma of spontaneous origin (NFSA), which arose in a C3Hf/Bu female mouse, was kept in liquid nitrogen, of which the 13th generation transplant was used throughout these experiments. The procedure to make single cell suspensions from this tumour has been described previously (Ando *et al.*, 1979). Briefly, tumours were removed and minced with scissors. The mince was then added to a beaker containing 30 ml Solution A (8.0 g NaCl, 0.4 g KCl, 1.0 g glucose, and 0.35 g $NaHCO_3$ in 1 litre $H_2O$) with 0.4% trypsin, 0.08% pancreatin, and 10 mg DNase. The first agitation (5 min at 37°C) was discarded because of the presence of dead cells and debris. The second agitation (15 min) was passed through a Swinney filter and resuspended with McCoy's 5A medium containing 5% foetal calf serum (FCS). Viability of these cells was always >95%. For lung colony assay, $\sim 2\times10^5$ viable cells suspended in McCoy's 5A medium with 5% FCS were injected i.v. For $TD_5$ assays, appropriate serial dilutions of cell suspensions were prepared and injected.

0007-0920/83/010073-07 $01.00

© The Macmillan Press Ltd., 1983

*Radiation*

For partial irradiation of the intestine, a $^{137}Cs$ $\gamma$-ray unit with a dose of 9.7 Gy min$^{-1}$ (two-opposed sources) was employed. Under Nembutal anesthesia (58.8 mg/kg), specified parts of the gut were exteriorized and placed within the radiation field (3 cm diameter) with build-up material (MIX-D). The gut was immersed in saline throughout the period of irradiation.

For whole abdominal irradiation, we used $^{137}Cs$ units with dose rates of 0.9 Gy min$^{-1}$ or 9.7 Gy min$^{-1}$, and a 250 KVp X-ray machine (HVL 1.2 mm Cu, FSD 60 cm) with a dose rate of 0.7 Gy min$^{-1}$. For the X-ray beam, 3 mm of lead was used to shield the head, chest, and legs. This resulted in a dose to the chest measured by TLD of $<8\%$ of the dose specified to the abdomen.

*Caecectomy*

Under Nembutal anesthesia, the caecum was exteriorized and surgically removed, care being taken not to injure the mesenteric blood vessels. Mice received no antibiotics, and 80% survived to be used in experiments 2 to 3 months later.

*Antibiotic therapy*

In experiments to investigate the role of microbial flora on AIRIM, antibiotics were administered via the drinking water continuously from 2 days before abdominal irradiation until the day of sacrifice. The following cocktail was used:

| | |
|---|---|
| chlortetracycline | 2 g l$^{-1}$ |
| gentamycin | 40 mg l$^{-1}$ |
| kanamycin | 1 g l$^{-1}$ |

*End points*

*(1) Lung colony assays.* Mice were injected i.v. with $\sim 2 \times 10^5$ NFSA tumour cells, and killed 11 days later. Their lungs were removed, and fixed in Bouin's solution, and the number of tumour nodules on the surface of the lungs were counted macroscopically.

*(2) $TD_{50}$ assays.* The number of tumour cells to raise tumours in 50% of mice, was determined by the serial dilution method. In i.p. challenge experiments, mortality was scored over a period of 3 months. For s.c. challenge, 4 sites were injected in each mouse, and tumour takes were scored by palpation for a period of 2 months.

*(3) Tumour volume measurements.* Three diameters (a, b, and c) of each tumour were measured by a caliper in mm. The formula $v = \pi/6$ abc gave the tumour volume in mm$^3$.

*Statistical analysis*

Student's $t$ test was employed and $P$ values $<0.05$ were considered significant.

### Results

*Localization of target tissue*

To localize the target tissue that had to be irradiated to exert AIRIM various parts of the gut were exteriorized and irradiated. As most of the large intestine is attached to the posterior abdominal wall, only the small intestine (jejunum and ileum), and caecum could be exteriorized. Immediately after irradiation, the gut was returned to the peritoneal cavity and the wound closed with surgical staples. Alternatively, peritoneal exudate cells (PEC), harvested by a single washing of the peritoneal cavity with 10 ml of physiological saline, were irradiated *in vitro* and injected i.p. back into the mice immediately after irradiation. Mice were injected i.v. with $2 \times 10^5$ NFSA cells 7 days after irradiation.

In the first experiment, either the small intestine (jejunum and ileum) or PEC were irradiated with 12 Gy $\gamma$-rays (Table IA). Neither of these procedures exerted AIRIM; the stress of sham irradiation of the small intestine slightly increased the number of lung colonies found (group 1 *vs* 2). In the second experiment, either the small intestine or the caecum was irradiated (Table IB). Irradiation of the small intestine again did not reduce the number of lung colonies but caecal irradiation did so significantly (Group 4, $P<0.001$). Irradiation of both small intestine and caecum also reduced the number of lung colonies (Group 2), presumptively identifying the caecum as a target organ for AIRIM.

The question as to whether the caecum is the only target or whether other parts of the large intestine were also involved was answered by the following experiments (Table IC). In the first, the caecum and small intestine were exteriorized and *shielded* while the abdomen, with the remainder of the large bowel *in situ*, was irradiated (i.e., irradiation of the "empty abdomen"). The number of lung colonies was not significantly reduced by this procedure (Group 1 *vs* 5). However, when the exteriorized small intestine and caecum were irradiated in addition to the "empty abdomen", the number of lung colonies was significantly reduced (Group 6, $P<0.001$) compared with sham-irradiated controls.

**Table I** Evidence that the caecum is the target organ in AIRIM

| | Irradiated organ | No. of mice | Number of lung[1] colonies (mean ± s.e.) | % of control | *P* value |
|---|---|---|---|---|---|
| A | 1. No irradiation | 7 | 128.4 ± 11.2 | — | |
| | 2. Small intestine sham | 6 | 172.0 ± 16.5 | 100.0 | |
| | 3. Small intestine | 10 | 124.0 ± 15.3 | 72.1 | NS |
| | 4. PEC sham | 8 | 131.3 ± 7.7 | 100.0 | |
| | 5. PEC[2] | 7 | 135.6 ± 19.0 | 103.3 | NS |
| B | 1. Small intestine + caecum sham | 8 | 127.4 ± 14.1 | 100.0 | |
| | 2. Small intestine + caecum | 8 | 58.9 ± 9.3 | 46.2 | < 0.005 |
| | 3. Small intestine | 8 | 112.4 ± 14.0 | 88.2 | NS |
| | 4. Caecum | 8 | 22.4 ± 4.3 | 17.6 | < 0.001 |
| C | 1. Small intestine + caecum sham | 10 | 65.6 ± 8.5 | 100.0 | |
| | 2. Small intestine + caecum | 8 | 14.9 ± 1.9 | 22.7 | < 0.001 |
| | 3. Small intestine | 9 | 48.0 ± 6.8 | 73.2 | NS |
| | 4. Caecum | 8 | 21.7 ± 2.7 | 33.1 | < 0.001 |
| | 5. Abdomen except small intestine + caecum | 8 | 51.6 ± 12.1 | 78.7 | NS |
| | 6. Abdomen and exteriorized small intestine + caecum | 8 | 14.7 ± 3.4 | 22.4 | < 0.001 |
| D | 1. No irradiation | 8 | 144.4 ± 9.5 | 100.0 | |
| | 2. Whole abdomen | 7 | 11.4 ± 3.1 | 7.9 | < 0.001 |
| | 3. No irradiation (post-caecectomy) | 7 | 112.7 ± 14.5 | 100.0 | |
| | 4. Whole abdomen (post-caecectomy) | 6 | 128.2 ± 23.0 | 113.8 | NS |

[1] All mice received $1.8 \times 10^5$ NFSA cells i.v. 7 days after irradiation with 12 Gy $^{137}Cs$ $\gamma$-rays.
[2] PEC were irradiated *in vitro* with 12 Gy $^{137}Cs$ $\gamma$-rays, and immediately reinjected i.p.
NS = Not significant.

Finally mice whose caecum had been surgically removed were found *not* to respond to abdominal irradiation (Table ID, Group 3 *vs* 4) while age-matched controls clearly did so (Group 1 *vs* 2). Thus, the caecum was established to be the essential target organ for AIRIM.

### *Test for AIRIM in germ-free mice or antibiotic treated mice*

To determine whether the microbial flora of the caecum was implicated in the mechanism of AIRIM, the following experiments were performed. In the first, regular SPF mice were compared with germ-free animals. Abdominal irradiation with 10.2 Gy X-rays was followed 7 days later with i.v. injection of $5 \times 10^4$ or $2 \times 10^5$ NFSA cells. The results, set out in Table II show that AIRIM was not observed in germ-free animals; in fact, the yield of lung colonies was slightly increased by abdominal irradiation.

The second experiment compared regular SPF animals against the same animals treated with antibiotics to achieve bacterial decontamination of the gut. Antibiotic therapy was administered via the drinking water using the combination described under **Materials and methods**. The results (Table III) showed substantial impairment of AIRIM (just short of statistical significance) in antibiotic-treated mice, supporting the hypothesis that enteric microorganisms were implicated in the effect.

**Table II** Lack of AIRIM in germ free mice

| Mice | Abdominal irradiation[1] | Number of lung colonies [mean + s.e. (number of mice)] $5 \times 10^4$ NFSA cells | % of control | $2 \times 10^5$ NFSA cells | % of control | *P* value |
|---|---|---|---|---|---|---|
| Regular SPF | – | 44.9 ± 6.7 (8) | | 138.9 ± 14.5 (7) | | |
| | + | 6.3 ± 1.4 (7) | 14.0 | 12.5 ± 2.4 (8) | 8.0 | <0.001 |
| Germ free | – | 41.6 ± 4.9 (5) | | 143.6 ± 16.6 (5) | | |
| | + | 59.6 ± 11.1 (5) | 143.3 | 170.6 ± 18.8 (5) | 118.8 | NS |

[1]Mice received 10.2 Gy, 250 kVp X-rays, 7 days before i.v. challenge.
NS = Not significant.

**Table III** Effect of antibiotics on AIRIM

| Mice | Abdominal irradiation[2] | Number of lung colonies [mean ± s.e. (number of mice)] $2 \times 10^5$ NFSA cells | % of control | *P* value |
|---|---|---|---|---|
| Regular SPF | – | 149.0 ± 14.5 (7) | | |
| | + | 29.1 ± 6.2 (7) | 19.5 | <0.001 |
| Antibiotic treated[1] | – | 141.9 ± 16.9 (7) | | |
| | + | 96.7 ± 13.7 (7) | 68.1 | $0.05 < P < 0.1$ |

[1]As described in Materials and methods.
[2]Mice received 12 Gy $^{137}Cs$ $\gamma$-rays, 7 days before i.v. challenge.

**Table IV** Effect of split-dose abdominal irradiation on lung colony formation

| | Abdominal irradiation: Dose (Gy) 250 KVp X-rays | Time (day) | No. of lung colonies (mean ± s.e.) 8 mice/group | % of control | *P* value |
|---|---|---|---|---|---|
| 1. | No treatment | | 116.1 ± 13.2 | 100.0 | |
| 2. | 10.2 | −7 | 14.6 ± 3.1 | 12.6 | <0.001 |
| 3. | 5.1 | −7 | 104.4 ± 14.7 | 89.6 | NS |
| 4. | 5.1 | −7 | | | |
| | + | | 11.5 ± 2.9 | 9.9 | <0.001 |
| | 5.1 | −5 | | | |
| 5. | 5.1 | −14 | | | |
| | + | | 104.0 ± 6.2 | 89.6 | NS |
| | 5.1 | −7 | | | |

All mice received $2 \times 10^5$ NFSA cells i.v. on day 0.
NS = not significant.

*Split dose irradiation*

In our previous report (Ando *et al.*, 1980), we showed that 12 Gy $^{137}Cs$ $\gamma$-rays (equivalent to 10.2 Gy X-rays) significantly reduced lung colonies while 6 Gy (equivalent to 5.1 Gy X-rays) had no apparent effect. We therefore examined the temporal relationship of splitting an "effective dose" (10.2 Gy X-rays) into 2 individually "noneffective doses" (5.1 Gy + 5.1 Gy). In a preliminary experiment, 2 doses of 5.1 Gy were separated by an interval of either 2 days (7 and 5 days before i.v. challenge with $2 \times 10^5$ cells) or 7 days (14 and 7 days before challenge) (Table IV). The number of lung colonies with the first schedule (Group 4) was reduced as much as that following a single dose on day-7 (Group 2). However, a 7-day split resulted in no reduction of lung colonies at all (Group 5).

The fact that the efficacy of 2 individually noneffective doses depended on the time interval between the doses led us to perform a detailed time course study. All mice except the control group received one dose of 5.1 Gy to the abdomen 7 days before i.v. challange with $2.2 \times 10^5$ NFSA cells. Each group received additional abdominal irradiation (another dose of 5.1 Gy) either 2–11 days before, or 2–6 days after the fixed dose. As seen in Figure 1, mice receiving the variable dose 2 days before, or 2 days after the fixed dose showed AIRIM while those receiving the variable dose 6–11 days before, or 4–6 days after the fixed dose did not. Mice that received their second dose 6 days after the fixed dose (i.e., 1 day before i.v. challenge) developed more lung colonies than unirradiated controls.

**Figure 1** Time course for split-dose abdominal irradiation. An "effective" dose of 10.2 Gy (X-ray) was split into 2 individually noneffective doses of 5.1 Gy. All mice (7–8 per group) received one dose 7 days before i.v. injection of $2.2 \times 10^5$ NFSA cells; the second dose was delivered at varying times as indicated by (—▼—). Results are plotted as the ratio between the number of lung colonies in irradiated mice compared with that in untreated controls, and errors indicate the 95% confidence limits of this ratio. The time course for inhibition of lung metastases from a single dose of 12 Gy $\gamma$-rays to the abdomen is shown for comparison (———).

*I.p. and s.c. challenge*

One of the unanswered questions regarding AIRIM was whether abdominal irradiation would reduce tumour transplantability by other than the i.v. route. We therefore studied the transplantation kinetics of tumour cells injected either s.c. or i.p. into mice that had received 12 Gy $\gamma$-rays abdominal irradiation 7 days previously. $TD_{50}$ values with 95% confidence limits for i.p. challenge were 97 cells (65–146) for untreated controls and 98 cells (55–177) for irradiated mice. With s.c. challenge, $TD_{50}$ values were 1750 cells (1196–2561) in untreated controls and 2616 cells (1824–3752) for irradiated mice, the difference not being significant.

*Effect of abdominal irradiation on spontaneous lung metastases*

Mice bearing NFSA tumours in the leg develop lung metastases, which can be observed 25 days after leg tumour transplantation (Figure 2). Mice with 7- or 14-day old leg tumours received abdominal irradiation (10.2 Gy X-rays) shielding the lungs and the leg tumour. The animals were sacrificed either 30 or 37 days after transplantation at which time the number of lung colonies seeded

**Figure 2** Spontaneous lung metastases in mice bearing NFSA tumours. The "primary" tumour growth curve following implantation of $5 \times 10^5$ NFSA cells into the right hind leg is plotted (●). Six mice from the group were sacrificed periodically and the number of visible lung metastases (○) was counted. Error bars indicate s.e.

**Table V** Effect of abdominal irradiation on spontaneous lung metastases

| Abdominal irradiation[1] | No. of animals | Size ($mm^3$) of leg tumour (mean ± s.e.) | P value | No. of spontaneous lung metastases[2] (mean ± s.e.) | P value |
|---|---|---|---|---|---|
| Experiment 1 | | | | | |
| 1. No irradiation | 5 | 4856 ± 189 | | 20.8 ± 3.8 | |
| 2. D7 | 7 | 3922 ± 192 | <0.05 | 7.4 ± 1.8 | <0.01 |
| 3. D14 | 7 | 4084 ± 310 | NS | 16.6 ± 7.3 | NS |
| Experiment 2 | | | | | |
| 4. No irradiation | 6 | 6261 ± 310 | | 163.7 ± 18.7 | |
| 5. D7 | 5 | 5690 ± 289 | NS | 87.0 ± 19.2 | <0.025 |
| 6. D14 | 8 | 5707 ± 160 | NS | 108.0 ± 16.3 | <0.05 |

[1]The abdomen was irradiated with 10.2 Gy, 250 KVp X-rays 7 (D7) and 14 (D14) days after transplantation of $5 \times 10^5$ NFSA cells into the right hind leg.
[2]Scored on Day 30 (Experiment 1) or Day 37 (Experiment 2).
NS = not significant.

**Table VI** Effect of abdominal irradiation on leg tumour growth

| Time of abdominal irradiation[1] | Tumour volume ($mm^3$) (mean ± s.e.) 10 mice/group | | | | P value |
|---|---|---|---|---|---|
| | D7 | D14 | D21 | D24 | |
| 1. −7D | 221.3 ± 11.9 | 1020.6 ± 59.7 | 2031.5 ± 87.9 | 3198.7 ± 101.7 | NS |
| 2. No irradiation | 234.3 ± 13.0 | 1042.4 ± 61.1 | 2233.3 ± 119.9 | 3474.7 ± 131.8 | |
| 3. +7D | 223.7 ± 16.7 | 838.4 ± 45.1 | 1830.4 ± 109.2 | 2750.1 ± 132.4 | <0.025 |

[1]10.2 Gy, 250 KVp X-rays 7 days before (1) or 7 days after (3) transplantation of $5 \times 10^5$ NFSA cells into the leg.
NS = not significant.

from leg tumours was counted (Table V). Mice receiving irradiation on day 7 developed significantly fewer metastases than did unirradiated controls ($P < 0.01$). Day 14 radiation also reduced lung metastases but the difference was not significant. It was noteworthy that the size of the leg tumours at sacrifice in the irradiated mice was also smaller. We confirmed this effect of abdominal irradiation on leg tumour growth in the following experiments. Mice received 10.2 Gy X-rays abdominal irradiation either 7 days before or 7 days after leg tumour transplantation. Tumours were measured by calipers once a week (Table VI). In mice receiving the irradiation 7 days after transplantation tumour volumes were consistently smaller than those in unirradiated controls. Abdominal irradiation 7 days before transplantation did not affect tumour growth. The scattered dose delivered to leg tumours from abdominal irradiation (<0.82 Gy) was not responsible for this retardation of growth as 1.2 Gy $^{137}Cs$ $\gamma$-rays given locally to day 7 leg tumours did not affect their growth (data not presented).

### Discussion

The experiments reported in this paper yield the following main conclusions:

(1) Irradiation of the caecum was essential for AIRIM.

(2) The effect apparently depended upon the presence of microbial flora in the intestine at the time of irradiation, since it was not seen in germ-free mice, and was inhibited by antibiotic therapy.

(3) Split-dose irradiation only slightly reduced the magnitude of AIRIM provided both doses were given within the time window of the effectiveness of single doses.

(4) Spontaneous lung metastases from primary leg tumours were also reduced by appropriately timed abdominal irradiation, though to a lesser extent than lung colonies from i.v. injected cells.

(5) Abdominal irradiation did not significantly increase $TD_{50}$ values for either s.c. or i.p. tumour-cell challenge, but did cause growth retardation of established s.c. tumours.

These observations lead us to believe that the mechanism of AIRIM is most likely related to transmigration of enteric microorganisms (in highest concentration in the caecum) as a result of radiation-induced denudation of the mucous membrane. By virtue of the presence of gram-negative bacteria, such transmigration would amount to an endogenous infusion of endotoxin. Endotoxin has long been known to induce haemorrhagic necrosis in established tumours, but is inactive when given immediately following tumour cell transplantation (Andervont, 1936).

Parr *et al.* (1973) investigated the effect of endotoxin administration *before* tumour cell challenge and found that its effect depended both on the timing of endotoxin injection and the route of tumour cell challenge. Thus, $10\,\mu g$ endotoxin given i.p. 3 or 7 days before i.p. injection of tumour cells yielded marked protection, but no protection was afforded by endotoxin given 1 day before tumour cells. For transplantation by the s.c. or i.d. routes, endotoxin was ineffective at all times. These results were interpreted as reflecting the accumulation of endotoxin-activated macrophages within the peritoneal cavity, but not in the skin or subcutaneous tissues.

We believe that the mechanism of AIRIM is most likely due to similar activation of pulmonary macrophages by appropriately-timed endogenous exposure to endotoxin. The requirement for each half of a split dose of irradiation to be given $7 \pm 2$ days before i.v. tumour cell challenge to provide protection is consistent with this hypothesis. Also consistent is our observation that the $TD_{50}$ for s.c. transplantation was unaffected by abdominal irradiation. The one discrepant result is the unchanged $TD_{50}$ for i.p. transplantation that could well be due to depletion of macrophage precursors by the abdominal irradiation.

Our demonstration of inhibition of spontaneous lung metastases by appropriately-timed abdominal irradiation may have two non mutually-exclusive components. On the one hand, the growth rate of established "primary" tumours in the legs of mice receiving abdominal irradiation was reduced (consistent with endotoxin administration), thus reducing the pool of tumour cells free to disseminate. On the other hand, activation of pulmonary macrophages may have inhibited the growth of cells subsequently seeded to the lungs. Consistent with this possibility, reduction of lung metastases was most marked when irradiation was given on Day 7 after late tumour implantation (i.e. before dissemination had occurred) than on Day 14.

Experiments are currently under way to test directly the endogenous endotoxin hypothesis with regard to the mechanism of AIRIM, and these will be reported later.

This investigation was supported in part by grants CA-17769 and CA-06294 awarded by the National Cancer Institute, Department of Health and Human Services.

Animals used in this study were maintained in facilities approved by the American Association for Accreditation of Laboratory Animal Care, and in accordance with current regulations and standards of the United States Department of Agriculture and Department of Health and Human Service, National Institute of Health.

We are grateful to Larry Wilborn and his staff for the supply and care of the C3Hf/Kam mice used in these experiments.

Germ-free animals were produced by one of the authors (Tsuneya Matsumoto).

### References

ANDERVONT, H.B. (1936). The reaction of mice and of various mouse tumours to the injection of bacterial products. *Am. J. Cancer*, **27**, 77.

ANDO, K., HUNTER, N. & PETERS, L.J. (1979). Immunologically nonspecific enhancement of artificial lung metastases in tumour-bearing mice. *Cancer Immunol. Immunother.*, **6**, 151.

ANDO, K., HUNTER, N. & PETERS, L.J. (1980). Inhibition of artificial lung metastases in mice by pre-irradiation of abdomen. *Br. J. Cancer*, **41**, 250.

PARR, I., WHEELER, E. & ALEXANDER, P. (1973). Similarities of the anti-tumour actions of endotoxin, lipid A and double stranded RNA. *Br. J. Cancer*, **27**, 370.